# The Influence of Playing Formation on Physical Demands and Technical-Tactical Actions According to Playing Positions in an Elite Soccer Team

**DOI:** 10.3390/ijerph18084148

**Published:** 2021-04-14

**Authors:** José Luis Arjol-Serrano, Miguel Lampre, Adrián Díez, Daniel Castillo, Fernando Sanz-López, Demetrio Lozano

**Affiliations:** 1 Health Sciences Faculty, Universidad San Jorge, 50830 Zaragoza, Spain; jlarjol@usj.es (J.L.A.-S.); miki_tauste@hotmail.com (M.L.); adri.diez.1991@gmail.com (A.D.); 2 Faculty of Health Sciences, Universidad Isabel I, 09003 Burgos, Spain; danicasti5@gmail.com; 3 National Sports Medicine Program (NSMP) Aspetar Orthopedics and Sports Medicine Hospital, 29222 Doha, Qatar; fsanz79@yahoo.es

**Keywords:** match analysis, performance analysis, playing system, physical performance, technical profiles

## Abstract

The aim of this study was to examine the differences in the physical demands and technical-tactical actions encountered by soccer players between two playing formations (1-4-2-3-1 and 1-4-4-2) for each playing position. Twenty-three professional male soccer players who played 31 official matches participated in this study. Players were classified according to their playing position: central defenders (CD), wide defenders (WD), central midfielders (CM), wide midfielders (WM), offensive midfielders (OM) and forwards (FW). The physical demands were collected as total distance (TD), distance covered in different speed thresholds, and number of accelerations and decelerations. Also, the technical-tactical variables were recorded. The results showed that the 1-4-2-3-1 playing formation demanded decelerations between 2–4 m·s^2^ (*p* = 0.027; ES = 0.26) in comparison with 1-4-4-2 for all players. Likewise, forwards (FW) and central midfielders (CM) registered higher physical demands playing with the 1-4-2-3-1 compared to the 1-4-4-2 formation. Regarding the technical-tactical actions, they showed differences between the playing positions of the two playing formations. The findings suggest coaches prescribe specific training programs based on the influence of the playing formation and playing position on the physical demands and technical-tactical actions encountered by players during official match-play.

## 1. Introduction

A number of techniques have been used to establish the physical profile of soccer players and the great technological advance allows an increasingly detailed analysis of physical activity, sports tactics and technique [1,2,3]. In the context of training and competition, global positioning system (GPS) data has been applied in order to measure, monitor and evaluate external load [4]. Aspects of players’ performance including speed, distances covered, as well as the numbers of accelerations and decelerations during training sessions and games has been analyzed [5,6]. Moreover, semi-automatic recording techniques through multi-video cameras have been used to measure and monitor the physical demands of the players, and also different technical-tactical outcomes, as individual players and as a team. Therefore, the semiautomatic recording techniques through multiple video camera systems most used by the major soccer leagues have been the Amisco^®^(Athletic, Nice, France) of France [7,8]; the ProZone^®^ (Prozone Sports, Leeds, England) from England [9]; since 2011, the Mediacoach system^®^(Mediapro, Madrid, Spain) in the Spanish Professional Football League (Liga de Fútbol Profesional, LaLiga™) [10]; and Wyscout (Chiavari, Italy) that manages the data of the most important European leagues: Ligue 1 (France), Bundesliga (Germany), SeriaA (Italy), Premier League (England), Eredivisie (Holland ) and Primeira Liga (Portugal) [11,12,13,14].

Activity profiles and physiological demands in soccer are intermittent by nature [8,15]. Players switch between short multidirectional efforts of high intensity and longer periods of low intensity activity [16,17]. However, the technical-tactical nature of soccer has shown the physical demands are multifactorial [18] and these demands on players change according to the specific player position and the related tasks on the field [8,19,20,21,22,23,24], the playing formation [22,23,24,25], scoreboard dynamics [26], playing home or away [7,26], players’ level [9,18,24], playing style [27,28], match momentum, 1st or 2nd half [9,17], age and maturity state [29], gender [30] or the course the season [19,31]. Nevertheless, most of the studies have used a traditional analytic approach, without taking into account the relationship between physical outcomes and technical-tactical actions. Physical performance could have an impact on the technical aspects and the tactical role. Moreover, it seems complicated to refute the impact of the tactical playing formation on team performance [16,19,32]. The change of team formations is one of the most efficient instruments for coaches to change and control the players´ behavior and thus directly influence game performance [25]. In the last years, the evolution of tactical systems has increased their dynamism instead of their inflexibility. In the Spanish “La Liga”, due to the predominance of possession-based team playing styles [33], the 1-4-4-2 diamond system has emerged with growing popularity, where the four midfield players are located in a rhombus formation. In this system, during offensive game phases, wide midfielders (WMs) play in a more central position, generating spaces for attacks by wide defenders (WDs) [34,35].

To date, to our knowledge, no study has focused the analysis of the 1-4-4-2 diamond playing formation. Thus, the aim of this study was to examine the differences in the physical demands and technical-tactical actions encountered by soccer players between two different playing formations (i.e., 1-4-2-3-1 and 1-4-4-2 diamond) for each playing position. 

## 2. Material and Methods

### 2.1. Experimental Design

The design used in this study was descriptive and based on an observational methodology applied to the acquired data. The data was obtained across a total of 31 official matches of the Spanish Second Division, through global positioning system (GPS) devices APEX pod and a WyScout ^®^(WyScout Spa, Chiavari, Italy) multiple-camera tracking system in order to analyze the physical demands and the technical-tactical actions encountered by soccer players using two different playing formations (1-4-2-3-1 and 1-4-4-2 diamond). Two UEFA-qualified coaches observed each of the games to verify that formation was consistent throughout the game. Furthermore, care was taken by the coaches to exclude any games that involved dynamic formation transitions [22].

### 2.2. Participants 

Twenty-three professional male soccer players (age: 25.1 ± 3.6 years; height: 180.3 ± 5.4 cm; body mass: 75.6 ± 6.4 kg; body mass index [BMI]: 23.2 ± 1.2), playing in the Spanish Second Division, were selected to participate in this investigation. Informed consent granted by the participants or the signature of their legal representatives (if under 18 years of age) was the study inclusion criterion, while performing other physical activities with overload that may influence the results of the study during participation and not respecting the training guidelines dictated in the study were exclusion criteria. All the players trained around 10 h per week and played an official match on weekends. Players were classified according to their playing position in two playing formations of the team (i.e., 1-4-2-3-1, *n* = 19 and 1-4-4-2 diamond, *n* = 12). Each player always played the same position. The characteristics of the participants are listed in Table 1. A total of 204 observations were made. The elite club authorized all researchers to use the data collected. This research was approved by the ethics committee of the Universidad San Jorge (Zaragoza, Spain) report 08-20/21. This investigation was performed in accordance to the Declaration of Helsinki (2013) and met the ethical standards for Sport and Exercise Science Research [36].

### 2.3. Physical Demands

The players’ physical demands were monitored using microsensor units containing an 18 Hz GPS and 600 Hz triaxial accelerometer (APEX pod accelerometer, MAPPS Technology and Bluetooth LE; STATSports, Newry, North Ireland). The system has shown great reliability in the data for total distance, high intensity running and other speed ranges [37]. Each player wore their own unit which was inserted into the manufacturer-provided vest which holds the receiver tightly between the scapulae. The total distance (TD), distance covered at different speeds, such as 14.4 km·h^-1^ (D > 14.4), 19.8. km·h^-1^ (D > 19.8) and 25.0 km·h^-1^ (D > 25.0), number of accelerations between 2–4 m·s^-2^ (Acc2-4) and above 4 m·s^-2^ (Acc > 4), number of decelerations between 2–4 m·s^-2^ (Dec2-4) and above 4 m·s^-2^ (Dec > 4) were registered as indicators of physical demands. These physical variables were previously used [38,39,40]. Data was downloaded post-training/match and analyzed using a customized software package (Apex Version 1.2, STATSports). 

### 2.4. Technical-Tactical Actions

Players’ movements were captured during matches by cameras positioned in each of the stadiums at roof level and analyzed using proprietary software to produce a dataset on each player’s technical-tactical performance [22]. The technical-tactical outcomes were registered using a WyScout^®^ (Chiavari, Italy) computerized multiple-camera tracking system validated analysis tool [12,14]. The outcomes were classified into three categories: (1) general indicators, (2) defensive indicators and (3) offensive indicators. The technical-tactical variables have been used in previous studies [41]. For this study, the variables selected were: game volume (GV), the sum of defensive indicators and offensive indicators, ratio interceptions-turnover (IT), the sum of general indicators; defensive volume (DV) the sum of all defensive indicators: number of interceptions (IN), number of opposing pitch interceptions (OPIN) clearances (CL); offensive volume (OV), the sum of all offensive indicators: total pass (TP), long pass (LP), short-medium pass (SMP), forward pass (FP), attack zone pass (AZP), goal shot (GS), crosses (CR) and dribbles (DR). Data for those players who did not play the entire match was excluded from further analysis.

### 2.5. Statistical Analysis

Data is presented as the mean ± standard deviation (±SD). Normal distribution and homogeneity of variances was tested using the Kolmogorov-Smirnov and Levene tests. All analyzed variables had a normal distribution, and parametric techniques were applied. A t-test for paired samples was used to analyze the differences of physical demands, and technical-tactical actions encountered by soccer players for each playing position and for all players between the 1-4-2-3-1 and 1-4-4-2 diamond playing formations. The level of statistical significance was set at *p* < 0.05. Practical significance was assessed by calculating Cohen’s effect size (ES) [42]. Thresholds for ES statistics <0.2 is unclear, <0.6 is small, <1.2 is moderate, <2.0 is large, >2.0 is very large, and >4.0 is extremely large [43]. A threshold value of 0.2 between-subject standard deviations was set as the smallest worthwhile change, and unclear effect was then based on the disposition of the confidence interval for the mean difference to this smallest worthwhile effect. Statistical analyses were performed using SPSS for Windows version 25.0 (SPSS Inc., Chicago, IL, USA). 

## 3. Results

The characteristics of the physical demands encountered by soccer players for each playing position and for all players using the 1-4-2-3-1 and 1-4-4-2 diamond playing formations are shown in Table 2. Table 3 shows that a 1-4-2-3-1 playing formation demanded higher Dec2-4 (*p* = 0.027; ES = 0.26, small) in comparison with 1-4-4-2 for all positions. Likewise, the player position FWhad a large effect for Dec2-4, while the other groups had only a trivial effect. In addition, CM covered higher TD, D > 14.4, and D > 19.8 (*p* = 0.03–0.038; ES = 0.92/1.32, from moderate to large), and performed lower Acc > 4 (*p* = 0.030; ES = 1.05, moderate) playing with the 1-4-2-3-1 compared to the 1-4-4-2 formation.

The descriptives of the technical-tactical actions encountered by soccer players for each playing position and for all players using the 1-4-2-3-1 and 1-4-4-2 diamond playing formations are shown in Table 4 and Table 5. The 1-4-4-2 playing formation demanded higher GV, OV, TP, SMP and FP (*p* =0.030; ES = 0.37–0.60) in comparison with 1-4-2-3-1 for all players. WD performed greater OV, TP and FP (*p* = 0.029–0.042; ES = 0.71–0.83, M) playing with the 1-4-4-2 compared to the 1-4-2-3-1 formation. CM performed obtained higher GV, OV, TP, LP, SMP and FP (*p* = 0.000–0.001; ES = 0.69–1.90, from moderate to large) playing with the 1-4-4-2 in comparison to the 1-4-2-3-1 formation. WM performed higher GV, DV and TP (*p* = 0.029–0.048; ES = 0.93–1.18, moderate) playing with the 1-4-4-2 compared to the 1-4-2-3-1 formation. OM performed larger GV, IN, OV, TP, SMP and FP (*p* = 0.004–0.040; ES = 1.62–2.30, large) playing with the 1-4-4-2 in comparison to the 1-4-2-3-1 formation. 

## 4. Discussion

The aim of this study was to examine the differences in the physical demands and technical-tactical actions encountered by soccer players between two different playing formations (i.e., 1-4-2-3-1 and 1-4-4-2 diamond) for each playing position. Our study is the first to analyze different dimensions in the demands of playing formation 1-4-4-2 diamond using professional players. The main findings were: i) physical demands and the technical-tactical actions differ between both playing formations and specific playing positions; ii) specific player positions analyzed inside the playing formations, require different physical demands in FW and CM and “different technical-tactical actions” for WD, CM, WM and OM, in both playing formations. Specifically, comparing the 1-4-2-3-1 and 1-4-4-2 diamond tactical formations, high differences between physical demands were not observed, differences were only seen in the Dec2-4 outcome. However, similar research concluded a 1-4-2-3-1 system implies a higher demand in decelerations (>3 m/s) when compared to other systems (1-4-4-2 and 1-4-3-3) [23]. The non-prevalence of conditioning differences between playing formations is aligned with the results shown by Bradley el al. [22], which indicates a statistically non-significant difference along three playing systems (1-4-3-3, 1-4-4-2 & 1-4-5-1) in total distance, high intensity (>14.4 km/h) and very high intensity (19.8 km/h), while Tierney et al. [23], found statistically significant differences between tactical formations (1-4-4-2, 1-4-3-3, 1-4-2-3-1, 1-3-5-2 and 1-3-4-3), with the playing formation 1-3-5-2 showing the highest condition demands (total distance, high intensity and high metabolic load). There were no differences observed (total distance, high intensity) between playing formations 1-4-4-2 and 1-4-2-3-1 [23], which are systems quite similar to the ones used in our current study. Having less numbers of players in the defensive line, could explain these differences, because midfielders during the matches have to cover a higher total distance, with a higher intensity, than central defenders [9,44,45]. 

Technical-tactical actions were different when comparing the two systems. It seems playing a 1-4-4-2 diamond formation, demands a higher participation in the offensive phase of the game, thus several differences were found in GV, OV, TP, SMP and FP outcomes in accordance with the results of Bradley et al [22], who found 1-4-4-2 and 1-4-3-3 systems show a larger number of passes than 1-4-5-1, which is tactically closer in nature to the 1-4-2-3-1 system analyzed in our study, because both play with the same number of midfielders. This could explain why possession-based playing teams showed more attacks and passes than direct play teams [28]. This could indicate a 1-4-4-2 diamond preference on possession playing style teams that require greater control of the game. 

However, playing position is the most important factor in explaining the differences in physical demand between formations. Analysis of positional data regarding physical demands, across formations, showed differences in FW and CM. A 1-4-2-3-1 playing formation resulted in a higher TD, D>14.4, and D>19.8 and less Acc>4 than 1-4-4-2 for CM. Higher Dec2-4 were found for FW in 1-4-2-3-1. No differences were shown between playing systems for WD, CD, WM, OM. Tierney et al. [23] found CM in 1-4-3-3 covered a larger total distance l (10,643 ± 1093 m) > 11%, than in 1-4-4-2. In contrast, Bradley et al. [22], reported no conditioning differences for midfielders in three systems (1-4-3-3, 1-4-4-2 and 1-4-5-1). However, analyzing high intensity action, based on ball possessions, showed an increase in high intensity actions without ball possession for midfielders in 1-4-5-1 compared to 1-4-3-3 and 1-4-4-2 [22]. Similar to these findings, differences in physical demands for CM could be produced in the defensive phase, due to CM in 1-4-2-3-1 (similar system as 1-4-5-1) are in the inner zone with only an OM ahead, while for CM of 1-4-4-2 diamond, 2 WM are located in closed positions and an OM also ahead, helping in the defensive phase, reducing the number of running and defensive movements to CM. These results disagree with Tierney et al. [23], where 1-4-2-3-1 produced lower deceleration demands for FW [23]. Despite studies presenting different formations, the tactic role of a player seems a powerful determinant of the physical performance [16]. The role of only one FW, can be focused on short efforts, through multidirectional movements in the area to score. This could be the reason for more Dec>2-4 in 1-4-2-3-1 than 1-4-4-2 diamond, where FW are not fixed, moving to free spaces in the flanks, generated by the location of the WM in the inner areas. 

The analysis of positional data in relationship with technical-tactical across formations showed some differences in WD, CM, WM, OM. A 1-4-4-2 playing formation demands higher values in OV, TP and FP for WD; GV, OV, TP, LP, SMP and FP for CM; GV, DV and TP for WM; GV, IN, OV, TP, SMP and FP for OM. No differences were found in the rest of the positions between systems. Bradley et al. [22] found the defender in a 1-4–4–2 system performed a greater number of passes and another technical actions than the defender in another playing formations (1-4–3–3 and 1-4–5–1). However, there were no differences between CD and WD in this research. Offensive tactical aspects could explain the results for WM. In 1-4-4-2 diamond, there are no WM located in the flanks, so perhaps this fact requires WD to be more present in offensive actions in the creation area to keep ball possession and going forward. To our knowledge, this is the first study that compares the role of OM in two different tactical formations. CM and OM, showed similar results, with a higher volume of offensive play in a 1-4-4-2 diamond formation. 

These findings are likely related to more influence in the attack for both positions. Although physical demands are lower, CM in 1-4-4-2 diamond has a greater influence in the offensive play, maybe with a major responsibility in attack, undertaking a key role in the distribution of the ball into the creative zone, and therefore doing more TP, LP, SMP and FP. In the offensive phase of 1-4-2-3-1, both CM must share the role in the creative zone, while in 1-4-4-2 diamond, This task is well defined by the presence of only one CM. Konefał et al [46], showed OM needed to be the more multiskilled player in order to adapt to play in any position in the formation [47]. Our results agree with these authors, because playing with one forward in 1-4-2-3-1 system, could cause OMs to play in a similar way as the FW, with more participation in goalscoring areas and less influence in the creative zone than OM in 1-4-4-2 diamond. This key role is focus on build up and organizing play, with higher numbers of OV, TP, SMP and FP. These could be the cause of the differences in the tactical-technical markers. Hughes et al, [48] reported WM playing in an opened position performed more assistant passes, after high intensity running, than the players in other positions, due to most of these efforts finishing in the wide zones of the opponent field [48]. In line with this, our results showed an opened field presence WM in 1-4-2-3-1, had a bigger impact in the goalscoring zones, because there is a non-significant increase of CR and GS, rather than WM in 1-4-4-2 diamond, however a closed position of WM in 1-4-4-2 diamond, demands a higher responsibility in building up the play. 

This study is not exempt of limitations. The first limitation is related to the fact that playing systems of the opponent teams in every match were not taken into account. Since, it is not possible to control the high variability of each team at each moment of the game. Therefore, this limitation was solved by analyzing the stable game formations that the same team always performs. Secondly, the scoreboard dynamics or if the team is home or away are not considered, and both factors can impact play [7,49]. The interaction of contextual variables would make the analysis and interpretation of the results more complex, due to the high number of qualitative variables. Thirdly, despite measuring external load, no internal load values were reported, such as RPE and/or heart rate. These limitations will be considered in subsequent investigations.

### Practical Applications

In summary, the results of this study showed that physical demands and tactical-technical profile are influenced by the playing formation and playing position of the players and should be used to prescribe specific training plans in accordance to them, because the team playing formation determines the role, tasks and responsibilities of the players. Coaches and practitioners should adopt a position-specific approach to player conditioning in their teams. Moreover, due to these results, coaches could determine the profile of the next players to contract, based on the main tactical system to be used throughout the season or adapt this the tactical system to the characteristics of the available players. Therefore, it would be of interest to keep tracking physical profiles and technical-tactical markers of each playing position, related to different formations, using a similar large-scale evaluation sample [9,50]. 

Results in this study could help coaches and practitioners select the playing system according to the available players and taking into account the different physical and technical-tactical demands of the formations. Moreover, adopting a position-specific approach to player conditioning, would potentially be needed in teams. Further research could evaluate the physical demands linked with the movements as principles and sub-principles in the four phases of the game (attack, transition from attack to defense, defense, transition from defense to attack). 

The main practical approach for coaches is the knowledge of the implications of the technical actions during a season. This could determine the strategic behavior of the team and guide a successful model of play. Moreover, the technical-tactical actions implications per position in each system and style of play, is a clue to utilize them in training or matches, in order to enhance the performance and player selection, linked with the individual characteristics. 

## 5. Conclusions

The results of the present study demonstrate that playing formations influence physical demands and the technical-tactical actions of the players. Specific player positions in the analyzed tactical formations requires different physical efforts for FW and CM and different technical-tactical demands for WD, CM, WM and OM, in both playing systems. In addition, 1-4-4-2 diamond system have a better adaptation to teams with possession-based play styles, thus higher levels of OV and TP in different positions are required. 

## Figures and Tables

**Table 1:** Characteristics of the participants.

Group	Observations	Age (Years)	Height (cm)	Body Mass (kg)	Body Mass Index (BMI)
*Central Defenders (CD)*	48	26.8 ± 3.5	187.6 ± 2.7	83.3 ± 2.7	23.4 ± 0.9
*Wide Defenders (WD)*	44	24 ± 1.8	177.8 ± 5.7	70.5 ± 4.8	22.3 ± 0.7
*Central Midfielders (CM)*	28	26 ± 1.4	177.5 ± 6.4	73.4 ± 5.3	23.5 ± 0.1
*Wide Midfielders (WM)*	27	24 ± 5.5	178.3 ± 4.7	72.5 ± 8.2	22.8 ± 2
*Offensive Midfielders (OM)*	28	23 ± 1.4	177 ± 2.8	71.8 ± 6.2	22.9 ± 1.3
*Forwards (FW)*	29	25.8 ± 4.2	180.3 ± 5.4	78.2 ± 2	24.1 ± 0.9
*Total*	204	24.4 ± 1.1	179.8 ± 4	74.9 ± 4.8	23.2 ± 0.6

**Table 2:** Descriptives of the physical demands (mean ± SD) encountered by soccer players for each playing position and for all players using the 1-4-2-3-1 and 1-4-4-2 diamond playing formations.

	CD		WD		CM		WM		OM		FW		All	
	1-4-2-3-1	1-4-4-2	1-4-2-3-1	1-4-4-2	1-4-2-3-1	1-4-4-2	1-4-2-3-1	1-4-4-2	1-4-2-3-1	1-4-4-2	1-4-2-3-1	1-4-4-2	1-4-2-3-1	1-4-4-2
TD	10,261 ± 552	10,250 ± 494	10,713 ± 525	10,864 ± 439	11,517 ± 515	10,935 ± 290	11,682 ± 696	11,959 ± 614	12,529 ± 335	12,039 ± 645	11,039 ± 325	11,014 ± 610	11,114 ± 806	11,021 ± 809
D > 14.4	1667 ± 428	1555 ± 273	2309 ± 341	2270 ± 335	2517 ± 430	2051 ± 381	2458 ± 494	2871 ± 424	2811 ± 156	2778 ± 347	24 ± 2	24 ± 4	2282 ± 511	2218 ± 577
D > 19.8	405 ± 157	397 ± 104	800 ± 250	759 ± 181	565 ± 123	428 ± 168	694 ± 163	820 ± 197	714 ± 130	704 ± 142	732 ± 122	825 ± 223	629 ± 214	662 ± 252
D > 25	77 ± 62	68 ± 36	178 ± 89	177 ± 93	85 ± 59	48 ± 39	128 ± 85	152 ± 82	101 ± 73	127 ± 55	167 ± 35	242 ± 133	119 ± 79	143 ± 105
Acc2-4	165 ± 43	160 ± 32	168 ± 45	164 ± 38	161 ± 32	164 ± 37	162 ± 31	173 ± 44	223 ± 19	170 ± 50	178 ± 7	159 ± 22	169 ± 37	160 ± 42
Acc > 4	13 ± 11	14 ± 6	15 ± 9	19 ± 8	8 ± 6	14 ± 6	13 ± 8	15 ± 7	22 ± 10	17 ± 4	23 ± 4	23 ± 5	14 ± 9	17 ± 8
Dec2-4	146 ± 34	140 ± 25	152 ± 21	146 ± 30	170 ± 40	167 ± 36	151 ± 39	170 ± 40	191 ± 19	174 ± 26	152 ± 12	128 ± 18	158 ± 33	146 ± 38
Dec > 4	19 ± 13	23 ± 10	33 ± 21	35 ± 11	22 ± 15	25 ± 12	33 ± 17	34 ± 13	45 ± 10	39 ± 12	32 ± 7	31 ± 7	28 ± 17	30 ± 13

*Note*: CD: central defenders; WD: wide defenders; CM: central midfielders; WM: wide midfielders; OM: offensive midfielders; FW: forwards; TD: total distance (m); D > 14.4: distance covered above 14.4 km·h^-1^ (m); D > 19.8: distance covered above 19.8. km·h^-1^(m); D > 25.0: distance covered above 25.0 km·h^-1^(m); Acc2-4: number of accelerations between 2–4 m·s^-2^; Acc > 4: number of accelerations above 4 m·s^-2^; Dec2-4: number of decelerations between 2–4 m·s^-2^; Dec > 4: number of decelerations above 4 m·s^-2^.

**Table 3:** Mean differences (%) and effect sizes (ES; ± CL) of the physical demands (mean ± SD) encountered by soccer players for each playing position and for all players between the 1-4-2-3-1 and 1-4-4-2 diamond playing formations.

	CD		WD		CM		WM		OM		FW		All	
	Mean Differences (%)	ES	Mean Differences (%)	ES	Mean Differences (%)	ES	Mean Differences (%)	ES	Mean Differences (%)	ES	Mean Differences (%)	ES	Mean Differences (%)	ES
TD	0.1	0.02	1.4	0.30	−5.0	1.32 L **	2.4	0.37	−1.1	0.21	0.3	0.07	0.3	0.03
D > 14.4	−5.5	0.25	−1.8	0.12	−18.7	1.15 M *	17.9	0.79 M	−1.8	0.17	2.2	0.15	0.7	0.02
D > 19.8	1.6	0.04	−3.5	0.12	−28.5	0.92 M *	18.1	0.62 M	−2.1	0.09	10.0	0.35	6.7	0.1
D > 25.0	12.2	0.12	−1.2	0.02	−48.8	0.70 M	24.9	0.38 S	33.1	0.33 S	32.1	0.66 M	28.9	0.31 S
Acc2-4	−2.7	0.10	−3.7	0.10	0.9	0.04	0.8	0.02	−27.8	1.04 M	11.4	1.14 M	13.4	0.32 S
Acc > 4	20.6	0.20	45.8	0.51 S	161.1	1.05 M *	12.4	0.14	−18.5	0.36 S	2.6	0.11	38.2	0.37 S
Dec2-4	−2.8	0.13	−6.6	0.27S	−2.3	0.09	10.3	0.29 S	−9.7	0.68 M	16.2	1.37 L *	9.1	0.26 S *
Dec > 4	27.3	0.28 S	49.4	0.41S	14.0	0.13	5.7	0.07	−18.1	0.54 S	4.6	0.17	25.2	0.29 S

*Note*: ES: effect sizes; CD: central defenders; WD: wide defenders; CM: central midfielders; WM: wide midfielders; OM: offensive midfielders; FW: forwards; TD: total distance (m); D > 14.4: distance covered above 14.4 km·h^-1^ (m); D > 19.8: distance covered above 19.8. km·h^-1^(m); D > 25.0: distance covered above 25.0 km·h^-1^ (m); Acc2-4: number of accelerations between 2–4 m·s^-2^; Acc > 4: number of accelerations above 4 m·s^-2^; Dec2-4: number of decelerations between 2–4 m·s^-2^; Dec > 4: number of decelerations above 4 m·s^-2^. Standardized effect size thresholds: S: small; M: moderate; L: large. * Significant level set at *p* <0.05; ** Significant level set at *p* < 0.01.

**Table 4:** Descriptive of the technical-tactical actions (mean ± SD) encountered by soccer players for each playing position and for all players using the 1-4-2-3-1 and 1-4-4-2 diamond playing formations.

	CD		WD		CM		WM		OM		FW		All	
	1-4-2-3-1	1-4-4-2	1-4-2-3-1	1-4-4-2	1-4-2-3-1	1-4-4-2	1-4-2-3-1	1-4-4-2	1-4-2-3-1	1-4-4-2	1-4-2-3-1	1-4-4-2	1-4-2-3-1	1-4-4-2
GV	55.7 ± 12.6	58.2 ± 16.1	55.6 ± 10.1	63.7 ± 13.1	54.6 ± 10.2	80.7 ± 16.5	44.0 ± 10.1	60.9 ± 13.9	31.8 ± 5.8	54.3 ± 11.2	25.2 ± 9.5	32.0 ± 8.2	48.3 ± 14.9	57.7 ± 19.0
IT	1.2 ± 3.8	1.7 ± 2.8	-0.6 ± 3.8	-0.3 ± 3.5	2.1 ± 4.0	4.0 ± 3.7	-5.2 ± 8.6	0.7 ± 4.5	-1.0 ± 2.2	-2.1 ± 3.6	-8.0 ± 4.0	5.7 ± 2.9	-1.1 ± 5.4	-0.1 ± 4.5
DV	15.8 ± 1.8	15.1 ± 5.3	12.1 ± 4.1	11.5 ± 3.9	13.6 ± 3.9	15.0 ± 5.5	8.4 ± 5.0	12.8 ± 4.3	7.0 ± 4.2	8.4 ± 1.9	3.4 ± 3.0	5.7 ± 3.4	11.3 ± 5.3	11.9 ± 5.5
IN	5.4 ± 2.6	5.9 ± 2.8	5.7 ± 2.7	5.8 ± 2.5	8.3 ± 2.7	10.3 ± 4.2	5.6 ± 3.9	7.8 ± 3.8	2.3 ± 1.0	6.8 ± 2.3	1.2 ± 1.1	3.0 ± 1.9	5.4 ± 3.3	6.3 ± 3.5
OPIN	0.7 ± 0.7	0.6 ± 0.7	0.8 ± 1.3	0.6 ± 0.9	2.2 ± 1.1	2.6 ± 2.0	0.8 ± 0.8	1.6 ± 1.5	1.3 ± 0.5	2.1 ± 1.8	0.8 ± 0.5	1.0 ± 1.1	1.1 ± 1.1	1.1 ± 1.4
CL	6.2 ± 2.6	6.4 ± 3.1	3.1 ± 2.0	3.1 ± 1.7	2.8 ± 3.0	2.8 ± 1.8	1.8 ± 0.8	2.3 ± 2.1	0.0 ± 0.0	0.0 ± 0.0	0.8 ± 0.8	1.1 ± 1.4	3.0 ± 2.8	3.3 ± 2.9
OV	39.9 ± 11.8	42.6 ± 15.7	43.6 ± 9.5	52.2 ± 12.1	39.8 ± 12.5	65.7 ± 15.2	35.6 ± 7.6	46.0 ± 14.6	29.0 ± 5.0	45.9 ± 10.2	21.8 ± 8.7	26.3 ± 6.0	37.0 ± 11.8	45.3 ± 16.9
TP	39.2 ± 11.3	42.2 ± 15.3	42.0 ± 9.1	50.3 ± 11.9	39.6 ± 10.1	64.5 ± 15.4	32.4 ± 7.6	45.2 ± 13.2	27.0 ± 2.8	41.4 ± 9.9	18.4 ± 7.0	21.4 ± 6.3	35.7 ± 11.6	43.4 ± 17.2
LP	8.7 ± 4.3	7.0 ± 2.6	6.2 ± 2.8	7.1 ± 3.2	7.0 ± 1.8	9.3 ± 3.3	2.4 ± 1.7	4.6 ± 2.9	2.5 ± 1.7	2.8 ± 2.2	0.8 ± 0.8	1.5 ± 1.0	5.5 ± 3.7	5.6 ± 3.6
SMP	30.6 ± 11.0	35.3 ± 15.0	35.8 ± 8.8	42.9 ± 11.8	32.6 ± 8.9	55.2 ± 14.4	30.0 ± 7.3	40.6 ± 11.7	24.8 ± 3.9	38.6 ± 9.0	17.6 ± 7.0	19.9 ± 6.5	30.2 ± 9.9	37.7 ± 15.4
FP	30.1 ± 11.3	31.9 ± 11.1	24.6 ± 4.1	30.2 ± 7.8	28.2 ± 7.5	45.9 ± 11.7	18.8 ± 4.7	26.4 ± 9.0	15.3 ± 2.2	22.0 ± 5.4	9.4 ± 4.4	9.7 ± 4.4	23.2 ± 9.5	27.7 ± 13.2
AZP	0.8 ± 1.4	0.5 ± 0.7	9.6 ± 5.7	10.7 ± 5.5	4.2 ± 3.7	6.9 ± 5.2	11.8 ± 6.7	7.2 ± 4.7	10.5 ± 2.7	11.9 ± 6.2	7.8 ± 3.3	8.5 ± 3.9	6.5 ± 5.6	6.7 ± 5.6
GS	0.4 ± 0.7	0.4 ± 0.6	0.1 ± 0.3	0.2 ± 0.5	0.9 ± 0.9	0.5 ± 0.6	0.8 ± 0.8	1.3 ± 0.9	1.8 ± 1.3	1.9 ± 1.3	1.6 ± 1.1	2.8 ± 1.8	0.7 ± 1.0	1.0 ± 1.4
CR	55.7 ± 12.6	0.0 ± 0.2	2.4 ± 2.5	2.1 ± 1.7	1.6 ± 2.1	2.9 ± 3.1	1.8 ± 1.1	0.7 ± 1.0	2.0 ± 0.8	1.4 ± 2.6	0.2 ± 0.5	0.3 ± 0.4	1.3 ± 1.8	1.0 ± 1.8
DR	1.2 ± 3.8	0.4 ± 0.8	1.5 ± 0.9	1.7 ± 1.6	0.6 ± 1.0	0.7 ± 1.0	2.4 ± 2.6	1.7 ± 1.5	2.2 ± 1.0	3.4 ± 2.5	1.8 ± 1.1	1.9 ± 1.4	1.2 ± 1.4	1.4 ± 1.5

*Note*: CD: central defenders; WD: wide defenders; CM: central midfielders; WM: wide midfielders; OM: offensive midfielders; FW: forwards; GV: game volume; IT: ratio interceptions-turnover; DV: defensive. Volume; IN: interceptions; OPIN: opposing pitch interceptions; CL: clearances; OV: offensive volume; TP: total pass; LP: long pass; SMP: short-medium pass; FP: forward. pass; AZP: attack zone pass; GS: goal shot; CR: crosses; DR: dribbles.

**Table 5:** Mean differences (%) and effect sizes (ES; ± CL) of the technical-tactical actions encountered by soccer players for each playing position and for all players between the 1-4-2-3-1 and 1-4-4-2 diamond playing formations.

	CD		WD		CM		WM		OM		FW		All	
	Mean Differences (%)	ES	Mean Differences (%)	ES	Mean Differences (%)	ES	Mean Differences (%)	ES	Mean Differences (%)	ES	Mean Differences (%)	ES	Mean Differences (%)	ES
GV	3.3	0.1	13.7	0.60 S	46.6	1.78 L **	37.7	1.18 M *	70.0	2.30 VL **	32.7	0.62 M	25.0	0.60 M *
IT	−2.9	0.04	−13.3	0.20	45.2	0.65 M	-	-	-	-	-	-	5.6	0.08
DV	−0.35	0.35 S	−5.0	0.14	5.7	0.14	62.8	0.93 M *	33.7	0.49 S	31.5	0.36 S	22.6	0.39 S
IN	10.8	0.14	3.8	0.08	16.4	0.32 S	46.9	0.52 S	209.3	2.07 VL **	100.6	0.96 M	43.0	0.47 S
OPIN	1.6	0.04	−5.8	0.09	25.6	0.35 S	62.6	0.78M	109.6	1.40 L	66.7	1.4 L	17.8	0.32 S
CL	7.7	0.11	4.1	0.07	26.4	0.32S	40.1	0.51S	-	-	43.3	0.60 S	9.2	0.10
OV	4.5	0.12	19.1	0.73 M *	72.8	1.79 L **	24.0	0.65 M	56.4	1.90 L *	28.4	0.53 S	16.3	0.37 S **
TP	5.5	0.15	18.8	0.71 M *	63.0	1.90 L **	36.7	1.03 M *	49.1	1.86 L *	20.5	0.39 S	22.1	0.51 M **
LP	−16.8	0.32 S	13.8	0.27 S	27.8	0.69 M **	105.3	0.89 M	24.9	0.30 S	29.7	0.43 S	4.1	0.06
SMP	13.4	0.29 S	18.0	0.59 S	68.7	1.84 L **	33.1	0.95 M	53.3	1.83 L *	17.0	0.30 S	24.4	0.53 M **
FP	7.7	0.18	20.6	0.83 M *	62.7	1.80 L**	36.0	0.88 M	41.7	1.62 L *	4.1	0.06	24.3	0.42 S *
AZP	−36.0	0.50 S	15.3	0.20	74.0	0.59 S	−43.8	0.78 M	4.0	0.09	7.5	0.12	−7.0	0.08
GS	−5.6	0.11	-	-	−6.5	0.16	14.9	0.23 S	−16.2	0.40 S	55.2	0.75 M	−14.6	0.36 S
CR	-	-	−10.6	0.17	30.9	0.29 S	−30.8	0.89 M	194.3	2.53 VL	-	-	−5.6	0.09
DR	-	-	13.9	0.24 S	−10.2	0.20	−50.5	0.85 M	28.4	0.35 S	28.8	0.38 S	−0.3	0.01

*Note*: ES: effect size; CD: central defenders; WD: wide defenders; CM: central midfielders; WM: wide midfielders; OM: offensive midfielders; FW: forwards; GV: game volume; IT: ratio interceptions-turnover; DV: defensive. Volume; IN: interceptions; OPIN: opposing pitch interceptions; CL: clearances; OV: offensive volume; TP: total pass; LP: long pass; SMP: short-medium pass; FP: forward. pass; AZP: attack zone pass; GS: goal shot; CR: crosses; DR: dribbles. Standardized effect size thresholds: S: small; M: moderate; L: large; VL: very large. * Significant level set at *p* < 0.05; ** Significant level set at *p* < 0.01.

## Data Availability

The datasets generated and analyzed for this study can be requested by correspondence authors in epardos@usj.es and dlozano@usj.es.
